# Parametric analysis on the global design of flexible riser under different environmental conditions using OrcaFlex

**DOI:** 10.1371/journal.pone.0310360

**Published:** 2024-12-23

**Authors:** Chiemela Victor Amaechi, Harrison Obed Butler, Salmia Binti Beddu, Agusril Syamsir, Idris Ahmed Ja’e, Ahmed Reda, Xuanze Ju

**Affiliations:** 1 School of Engineering, Lancaster University, Bailrigg, Lancaster, United Kingdom; 2 Department of Construction Management, Global Banking School, Manchester, United Kingdom; 3 Institute of Energy Infrastructure, Universiti Tenaga Nasional, Jalan IKRAM-UNITEN, Kajang, Selangor, Malaysia; 4 DTU Energy, Danmarks Tekniske Universität (DTU), KGS Lyngby, Denmark; 5 Department of Civil Engineering, Ahmadu Bello University, Zaria, Nigeria; 6 School of Civil and Mechanical Engineering, Curtin University, Bentley, Western Australia, Australia; 7 Offshore Oil Engineering Co., Ltd., Engineering Company, Tianjin, China; Xi’an Jiaotong University, CHINA

## Abstract

The application of flexible risers has led to increased production of fluid contents in the marine industry. This paper presents the design challenges of a flexible riser subjected to internal pressure under deep-water conditions, at a water depth of 2000 m. Parametric variations with extensive dynamic analysis were carried out. The study highlights include the global analysis of lazy-wave configuration for the design of flexible risers, to understand the failure of flexible risers and application of hybrid configurations on flexible pipes. For the global analysis, the design of the riser was modelled in OrcaFlex by considering different sections and then analysed for the influence of effective tension, bending moment and environmental conditions. This riser model is multi-layered and was mainly subjected to the fluid pressure load and the environmental load. Model validations were performed with existing lazy-wave models. In the global design, the riser was assessed when connected to the vessel, but vessel motion was not included, additionally, three different environmental conditions were applied on the model. Also, the suitability of the adopted configuration for the proposed flexible riser was adopted considering it as a sustainable marine structure. Stress profile, tension profile and bending moment for the risers were presented and conclusions were made on the study. Some fatigue study is recommended in future study to be undertaken on the riser.

## 1. Introduction

**Fig 1:**
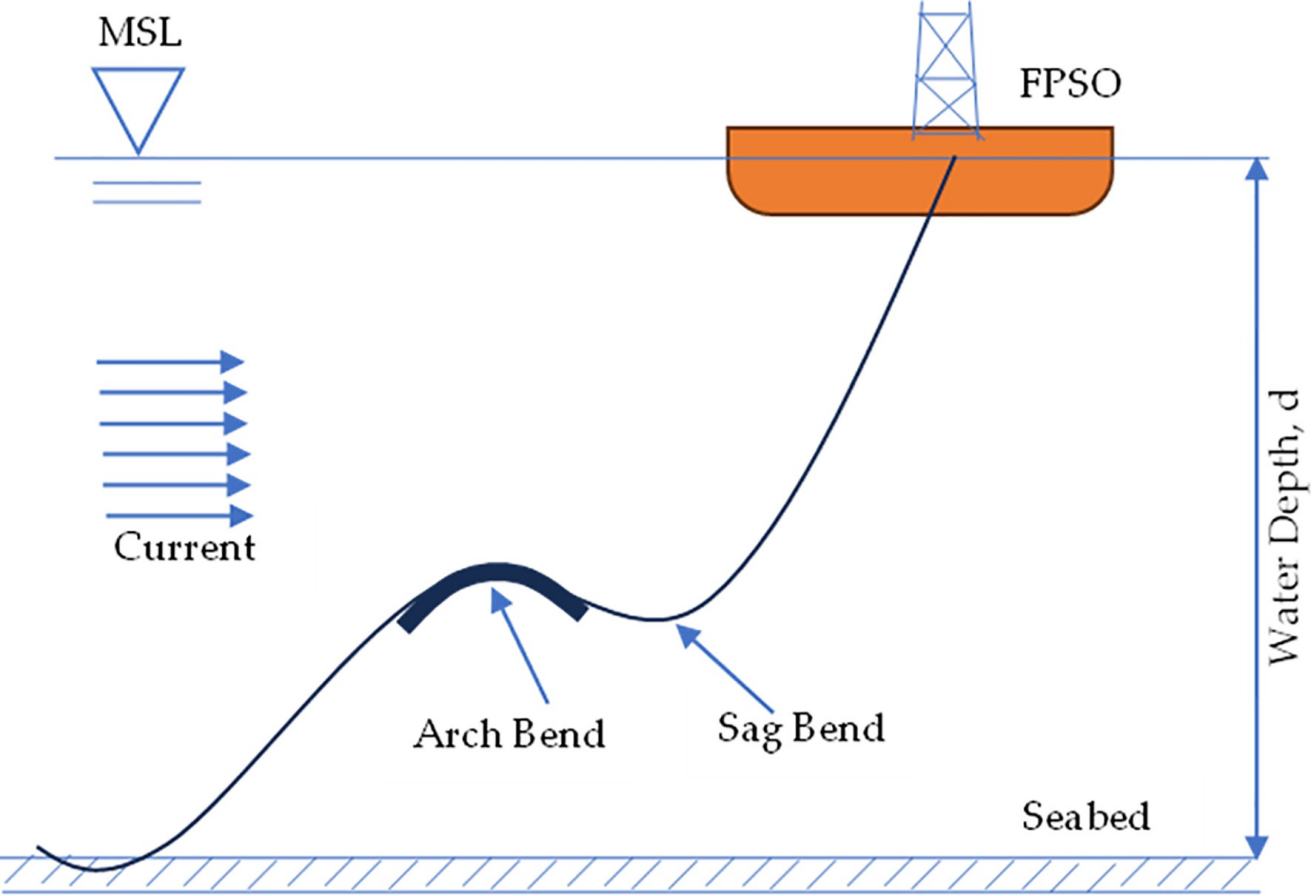
Typical configuration for marine riser under a lazy wave configuration.

The need to meet rising energy demands has led to increased production in the oil and gas industry [1, 2]. The quest for fossil fuels has spawned novel methods as well as concepts in the offshore extraction and transport of crude oil and LPG over the last half-century using various offshore platforms [3–6]. One of the components used in production of fluid includes offloading hose systems and other flexible risers [7–10]. These studies have been useful in developing a few industry handbooks on marine structures like flexible risers [10–12]. Some recent studies have been conducted on the fatigue of flexible risers [13–15]. Flexible riser systems have been used in Offshore West Africa, the Gulf of Mexico and North Sea, despite their origins in offshore locations with benign weather conditions such as offshore Nigeria, the Mediterranean, the Far East, and the pre-salt coast in Brazil. Despite the type of floating system, other important aspects include the design considerations and the coupling for the marine risers [16–19]. To that end, there are considerations for selection of the marine riser type, riser concept and the configuration that will be desirable for each design [20–25]. Also, there are different configurations that have been identified for marine risers such as Weight Added Wave (WAD) configuration [26, 27], Catenary Offset Buoyant Riser Assembly (COBRA) [28–32], and the hybrid riser systems including the use of composite risers and marine hoses [33–40]. Other configurations include Buoy for Supporting Lines (BSL) or Buoy Supporting Risers (BSR) [41–49], lazy-wave configurations [50–60] and flexible off-loading lines (OOL) [61–71]. Classification for marine riser concepts identified include the coupled riser concepts like Steel Catenary Risers (SCR), and Steel lazy wave riser (SLWR), while uncoupled riser concepts include Grouped Single Line Offset Riser (SLOR), Tethered Catenary Riser (TCR), Hybrid Riser Tower (HRT), Catenary Offset Buoyant Riser Assembly (COBRA), Single Hybrid Riser (SHR) and Buoyancy Supported Riser (BSR). Fig 1 shows a typical riser configuration.

While marine riser configurations may differ, the mean sea level (MSL) is theoretically taken as the top of the sea. The marine risers exhibit variations in performances, as displayed by the bending moments and effective tension profiles of the riser system. In principle, flexible risers offer significant benefits over other types of marine risers due to their high flexibility and lower bending stiffness. This permits them to be sufficiently flexible to be rolled, stored on reels or for installation at extended lengths. It also helps to ensure serviceability while enduring the harsh operating conditions encountered in subsea operations. Furthermore, flexible risers have facilitated and enabled the development of various places that would otherwise be unfeasible or prohibitively expensive if only traditional rigid pipes were available. Flexible risers, despite their advantages and capabilities, have disadvantages. Their intricate design helps them to survive dynamic and demanding conditions, but it also results in a significantly greater failure rate than for simpler all-steel pipes and risers. Flexible riser systems are used in connection with both FPSOs and other production platforms to carry fossil fuel products from the seabed to storage vessels as part of marine riser solutions, such as SLWR [72–83]. Some literature that presented typical patents on marine risers including flexible risers reflect developments on the inventions are available in recent reviews [40, 69, 70, 84–88] and related industry standards. Marine risers have complex behaviour including composite risers and flexible risers. Though, there are design limitations for each marine riser indicated in various industry standards (such as API, DNV, ABS, ISO). Both geometric and mechanical properties of flexible risers have been important in the design of the structure [89–91]. Consequently, there is a need for deeper understanding on the global design of flexible marine risers.

In this paper, the global design of flexible riser under different environmental conditions is presented. The design analysis of the flexible riser using lazy-wave configuration under waves and current was conducted with three (3) sea states. The study is introduced in Section 1, while Section 2 presents some background on the theory. The numerical tools for the modelling the global design include ANSYS AQWA and OrcaFlex. Section 3 presents the results and discussion, while Section 4 concludes the research. The proposed flexible riser model aims to provide extensive information on the stresses and forces experienced by various areas of the riser.

## 2. Materials and methods

In this section, the materials used for the design, and the research methodology for the study are presented.

### 2.1. Model description

**Fig 2:**
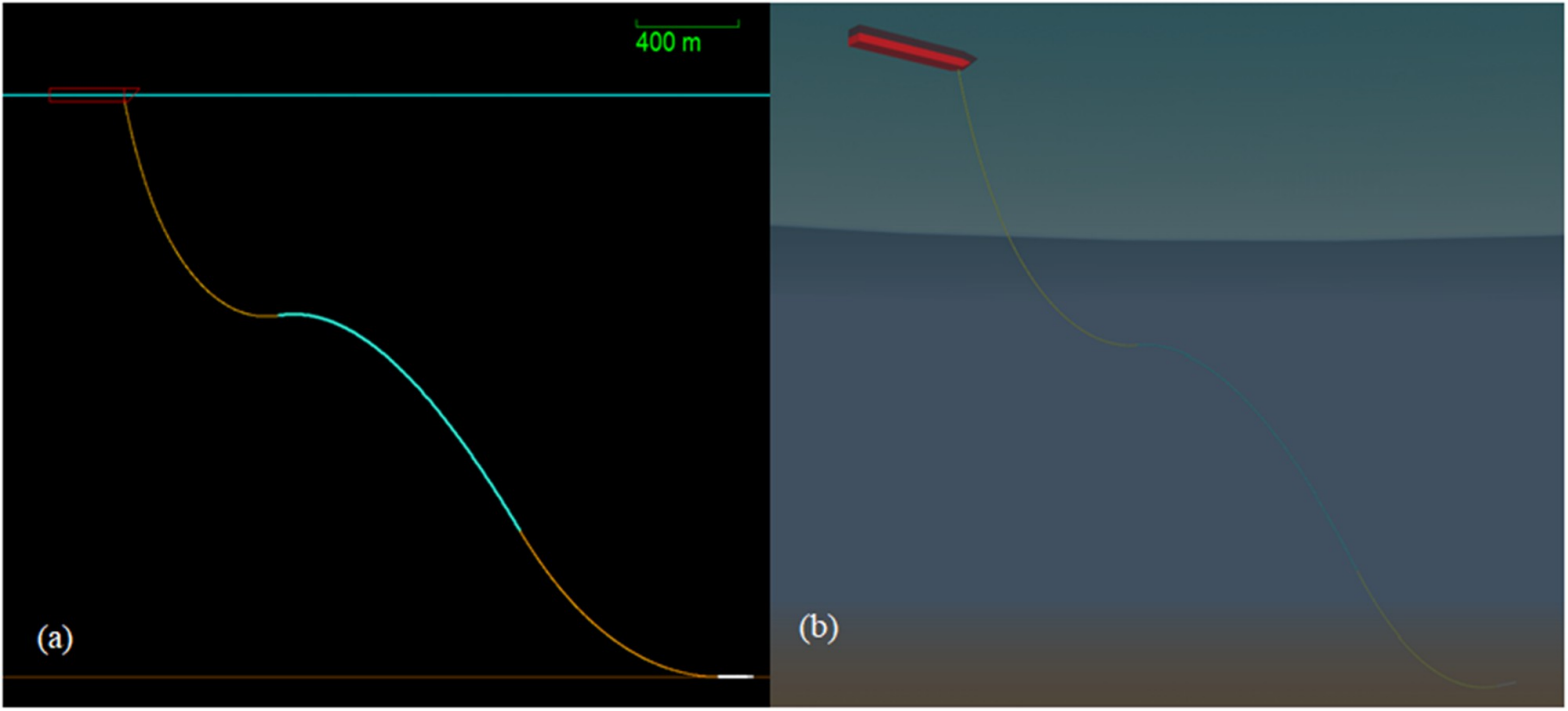
Model views of lazy-wave OrcaFlex Model, showing (a) unshaded and (b) shaded profile views.

The model used is a hybrid composite riser designed for a deep-water environment consisting of two model configurations combined, as depicted in Fig 2. This hybrid model is a mix of both configurations which provided the best mix for the multi-layered structure. The water depth considered in this design is 2000 m. The design of this flexible riser involves both the local design and global design, and is also considered attachment to an FPSO in a deep water conditions, in a free-hanging configuration. The local design was carried out first as a static and dynamic analysis, based on finite element modelling in ANSYS Structural R2 2020, and OrcaFlex 11.0f. This was coupled into the global analysis in ANSYS AQWA R2 2020 with analysis in OrcaFlex 11.0f.

This design consists of more than two layers, and is classified as a multi-layered structure. The water depth considered in this design is 2000 m, as presented in Table 1. This design was conducted by considering design for a typical deep-water flexible riser. In this study, the calculation for the tension of the flexible riser model looks at the effective weight of the riser based on the wall thickness used. This flexible riser is a novel multi-layered marine riser and has tensile armour reinforcements, among other layers. Additionally, the design of this riser was based on recommendations on industry standards. Details of the model are given in Sections 2.2–2.3.

**Table 1:** Parameters of the flexible riser.

Parameter	Value	Unit
Length of Riser	250	mm
Outer Diameter	0.335	m
Inner Diameter	0.269	m
Number of Layers	10	--
Water Depth	2,000	m

### 2.2. Design methodology

Certain design concerns are taken into account when designing this hybrid composite flexible riser. The design of this hybrid composite flexible riser is conducted in three stages. Stage 1 is the preliminary design at various local design sections along the riser’s length. Four crucial loads were considered which exclude the possibility of platform displacement when linked to the riser. These are top tension given to the riser to keep it vertical during loading, (as depicted in Fig 1, showing the top tension applied to the marine riser to maintain stability). Also acting on the riser structure are gravitational and buoyancy forces, internal pressure from the oil flow or natural gas being pumped up from the seabed, and lastly the hydrostatic forces. External forces acting on the riser, on the other hand, can be considered in both static and dynamic terms. A global examination of the riser’s response to effects, such as gravity, platform movement, buoyancy, hydrostatic pressures, and so on would constitute the second stage of design. This stage is used to identify the crucial places along the marine risers’ structure that encounter the most stress and other related forces that have a direct impact on the marine risers’ structure as well as its safety and integrity. This can further impact on the fluid flow containment. The third stage would be a stress analysis of these important locations in various scenarios involving various local loads operating on these points, as indicated by the prior global studies’ analysis. However, there are more analysis that need to be carried out to ensure structural integrity of the structure, aside from the ones presented in this paper. Finally, it is recommended that after successful pre-implementation modelling has been completed totally with good results that are well verified, the asset could be manufactured and implemented (though would be upon completion, and outside the scope of this study). In this investigation, the global design was employed to conduct the mechanical investigation on the behaviour of the riser structure. The global analysis was conducted using the OrcaFlex versions 10.3d and 11.0f, as detailed in subsequent sections.

### 2.3. Model setup

The model setup for the hybrid composite riser is designed for a deep-water environment consisting of the hybrid lazy-wave model configurations combined, as depicted in Fig 2. The numerical package utilised to simulate the model and offer the global analysis was OrcaFlex versions 10.3d and 11.0f. This software is designed primarily for maritime and offshore simulations, making it ideal for the needs of this study. OrcaFlex has been applied in various marine structure design and analysis [92–96]. The foundational modelling in Orcaflex was based on the theoretical aspects available on Orcina website [97, 98]. This software can be used to analyse and plot a range of riser parameters such as the bending moment and effective tension of the flexible riser. Furthermore, this software allows the simulated environment’s sea characteristics to be modified and tweaked. This enables riser analysis in a variety of offshore situations, including more severe scenarios like hurricanes, which necessitate more robust constructions. As a result, three distinct simulations were run, each with varying severity of environmental conditions.

The global analysis model consisted of a flexible riser coupled to an FPSO and the seabed in a sluggish-looking pattern called lazy-wave configuration. The flexible riser type was a 3390 m long line divided into six sections, one of which included floats. Table 2 provides section information on the flexible riser, while Table 3 gives the geometric data of the section considered for finite element modelling. The data were obtained from validated works in recent publications and material database (Granta and MatWeb). The riser’s connections and anchoring to the seabed were positioned in the FPSO’s bow. The FPSO was set up with zero degrees of freedom to ensure that it would remain stationary for the duration of the simulation. This simplification was made to facilitate a clearer understanding of the simulation. Fig 2 provides the global model of the hybrid composite flexible riser attached to the FPSO with more features of both the riser part and the FPSO for the model.

**Table 2:** Section information on the flexible riser.

*Riser Section*	*Section Length (m)*	*Target segment length (m)*	*Number of segments*
*Group 1 flexible 13”*	*1*,*050*	*2*.*0*	*525*
*Group 2 flexible 13” + Floats*	*1*,*250*	*4*.*0*	*312*
*Group 3 flexible 13”*	*530*	*1*.*0*	*530*
*Group 4 flexible 13”*	*500*	*1*.*0*	*500*
*Group 5 flexible 13”*	*40*	*1*.*0*	*40*
*Group 6 flexible 13”*	*20*	*4*.*0*	*5*

**Table 3:** Data on the flexible riser showing the mechanical and geometric properties.

*Layer No*.	*Layer Type*	*Material*	*Outer Radius (mm)*	*Inner Radius (mm)*	*Young’s Modulus (GPa)*	*Density (kg*.*m*^*-3*^*)*	*Poisson’s Ratio*
*1*	*Fabric Tape*	--	*55*.*75*	*55*.*25*	*0*.*6*	*800*	*0*.*30*
*2*	*Outer Tensile Armor Layer*	*F141*	*55*.*25*	*52*.*25*	*211*	*7870*	*0*.*29*
*3*	*Anti-friction Layer*	*Nylon 11*	*52*.*25*	*50*.*75*	*207*	*7850*	*0*.*30*
*4*	*Inner Tensile Armor Layer*	*F115*	*50*.*75*	*47*.*75*	*0*.*301*	*1040*	*0*.*29*
*5*	*Anti-friction Layer*	*Nylon 11*	*47*.*75*	*46*.*25*	*0*.*30*	*7850*	*0*.*30*
*6*	*Zeta Layer*	*---*	*46*.*25*	*40*.*05*	*---*	*---*	*0*.*29*
*7*	*Pressure Sheath*	*Nylon 12*	*40*.*00*	*35*.*10*	*0*.*28*	*1080*	*0*.*30*
*8*	*Carcass*	*AISI 304 Steel*	*35*.*10*	*31*.*60*	*205*	*7930*	*0*.*29*

Note: Material database (Granta and MatWeb); Sources: Ref. [89–91]

### 2.4. Hydrodynamic and environmental setup

**Fig 3:**
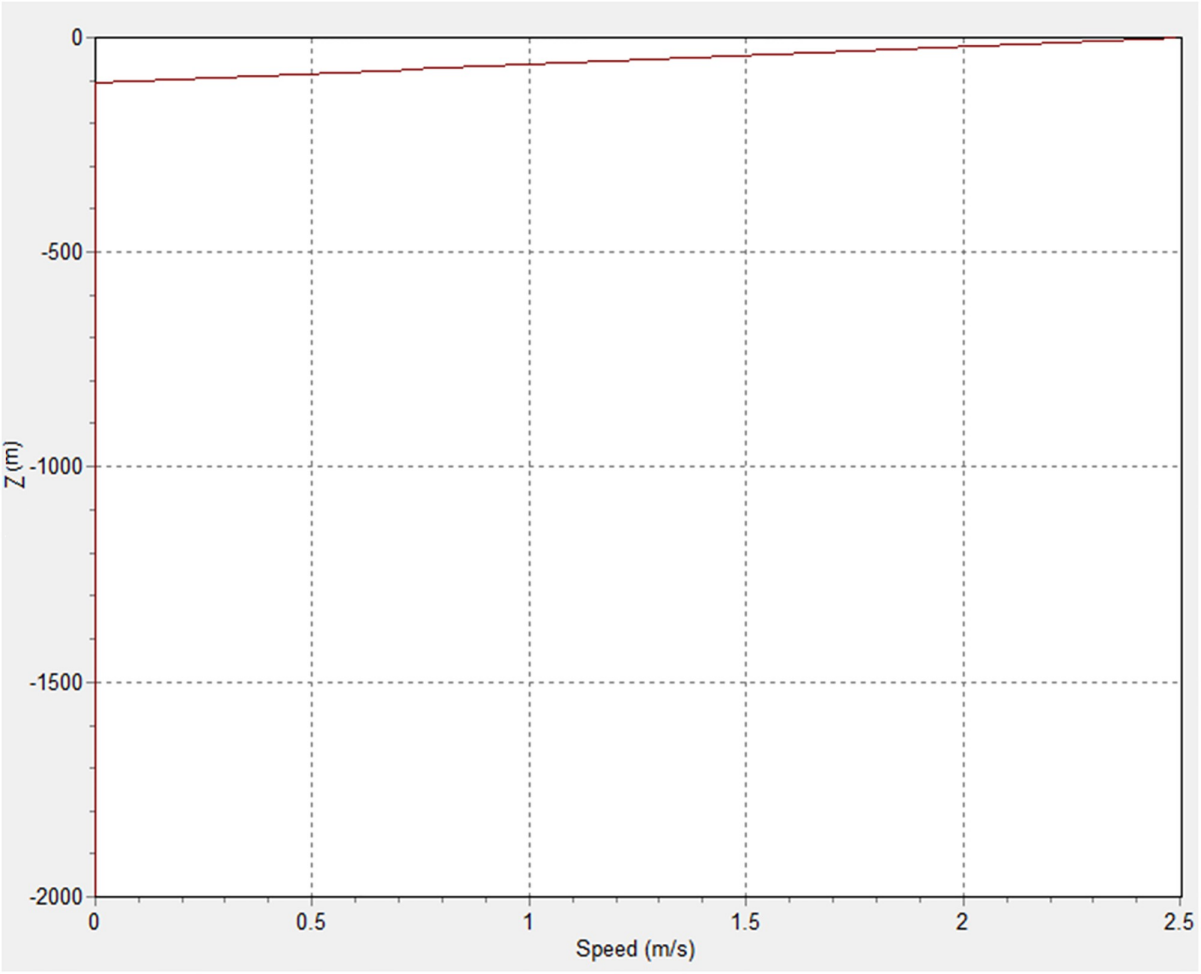
Current velocity profile in OrcaFlex 11.0f.

The choice on the environmental conditions was an incredibly important part of the simulation setup. Ensuring realistic and justified values is crucial in yielding useful results, especially for effective tension plots. It is noteworthy to state that the hybrid marine riser is modelled using industry guidelines in ABS [99], DNV [100–104], and API [105, 106]. However, other guidelines could also be utilised for the hybrid marine riser structure. One part of these conditions was the velocity profile of the sea current, for which three different values were chosen for each condition with reference to the API 2MET Standard for Derivation of Metocean Design and Operating Conditions [107]. Comparisons were made to the environmental states presented in industry specifications like the API bulletin [108] and DNV recommended practice [109] when selecting the case studies for the investigation. However, due to the limitation of the study’s scope, further work is suggested in the area by considering detailed hydrodynamic loadings. These industry standards consider the spatial correlation of sea states and wave scatter diagrams for different environmental conditions [107–109]. Though, the values for the surface current in this study as well as the 0-speed depth values were selected using API data. A velocity profile was then created by interpolating these two points creating a linear profile from surface velocity to 0-speed depth. Fig 3 shows the current profile for the model.

**Fig 4:**
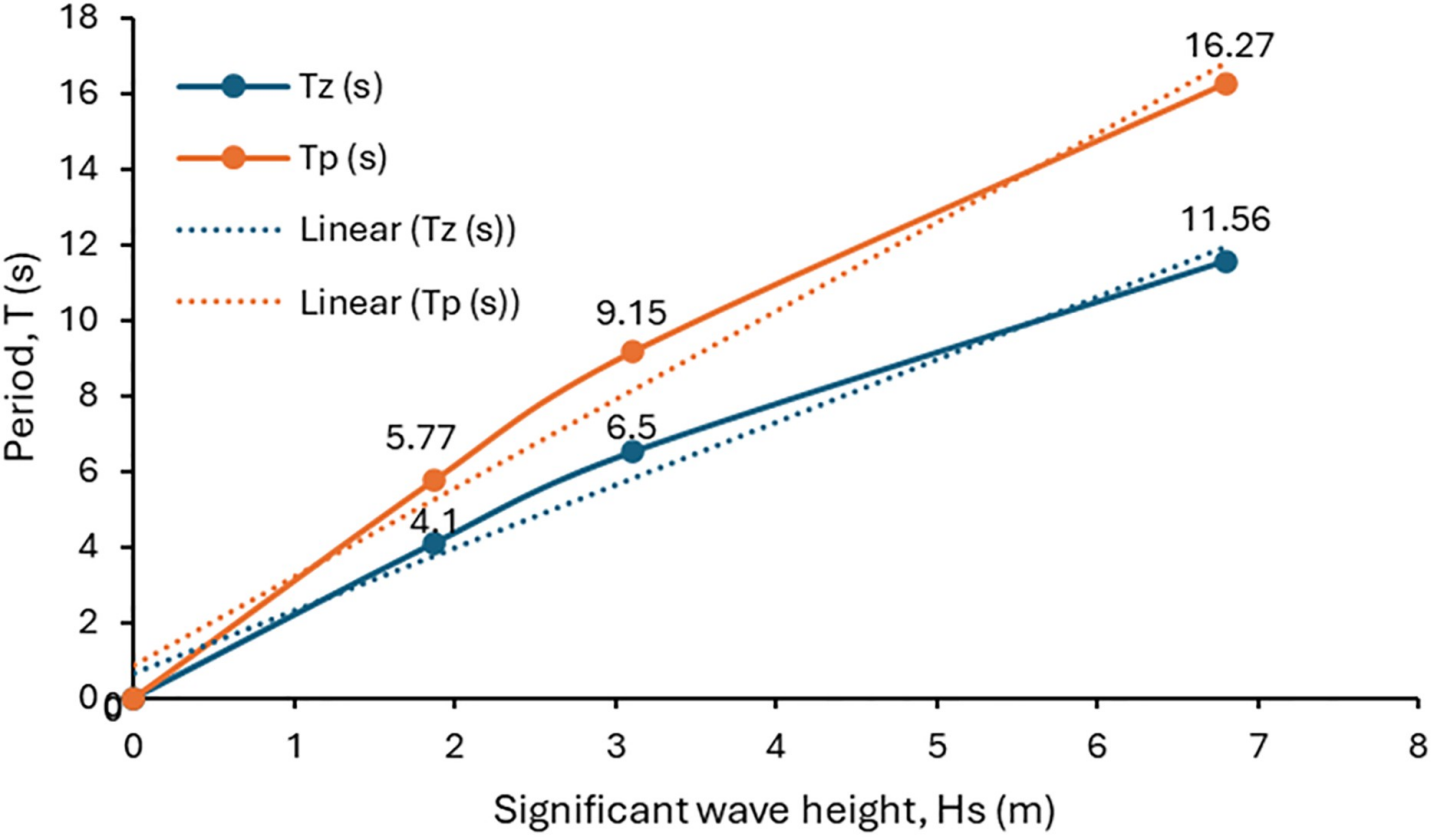
The scatter diagram for the Hs vs T profile.

With reference to this model, the parameters used for the waves were selected for operation, survival, and extreme conditions. Table 4 contains the detailed current profile for the 3 cases. Also, Table 5 provides the wave parameters for the model, which include significant wave height (Hs), zero crossing period (Tz) and peak period (Tp). However, the details of these conditions can be seen in the current and wave configuration in Figs 3 and 4, respectively. The scatter diagram for the Hs vs T (period) profile showing the spectral shape is presented in Fig 4.

**Table 4:** Current parameters for the 3 load cases.

Case	Surface speed (m/s)	0-speed depth (m)	Return period (y)
1	1.13	47.3	10
2	2.00	83.8	100
3	2.49	104.7	1000

**Table 5:** Wave parameters for the 3 load cases.

Case	*H*_S_ (m)	*T*_Z_ (s)	*T*_P_ (s)	Classification
1	1.87	4.10	5.77	Operation (Normal)
2	3.10	6.50	9.15	Survival (Squall)
3	6.80	11.56	16.27	Extreme (Hurricane)

### 2.5. Motions and loadings

An important aspect of the modelling is the consideration for the motion and the loadings. There are texts presenting fundamental theories on marine riser applications [5–10]. The FPSO is a structure that can be designed for different operations, controls and environments. The approach for the present study has been conducted in earlier parametric studies with flexible riser validation [89–96]. Relevant works have deployed OrcaFlex in modelling [92–97] as OrcaFlex tool is validated with its theory documentation [97, 98]. In addition, the development of marine components requires robust design considerations. These are seen in both industry standards on hybrid marine risers [99–106]. For the environmental loadings, the industry guidelines with relevant metocean data were used [107–109]. However, lesson learnt on various hybrid marine riser systems were also considered [33–40] as well as loadings from attachments on FPSOs [110–116].

**Fig 5:**
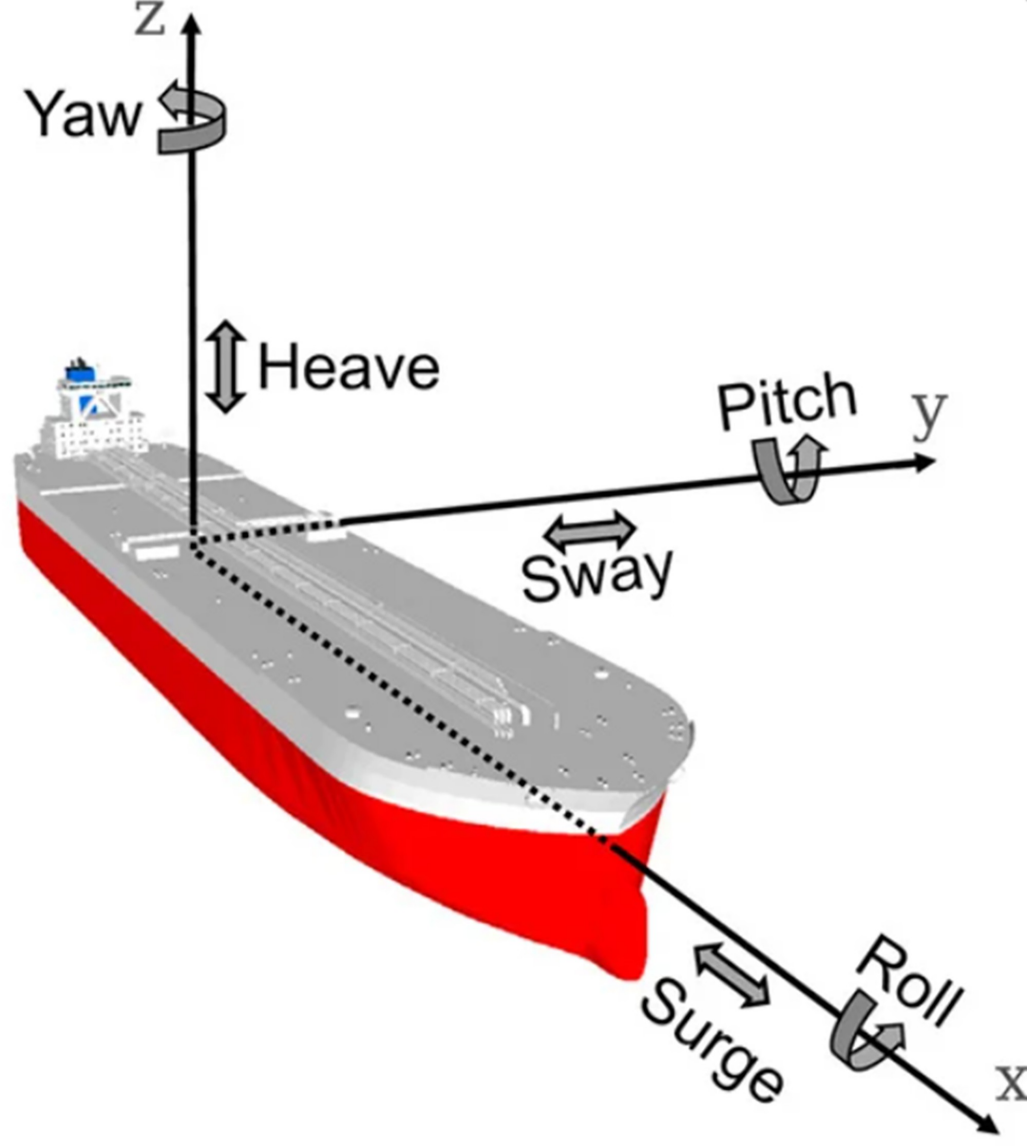
The 6DoFs of the FPSO vessel, showing the vessel’s motions along the three axes. (Image is adapted with permission from authors and reused as Open Access publication. Source: [110], Copyright year: 2021, Publisher: MDPI).

In principle, FPSO motions have been validated in available literature [94, 110–112]. Based on the motion, dynamic positioning (DP) and weathervaning are some applications based on motion of the FPSO. FPSOs can also be attached with marine risers and mooring lines [110–116]. The loads considered in OrcaFlex for this global design are given in Table 6. The configuration used is a marine riser system considers the application on an FPSO. The FPSO is a floating structure with 6DoFs. The direction for the motions on the FPSO is depicted in Fig 5.

**Table 6:** Loads considered on vessel motion in global design.

*Loads Considered*	*Loads not considered*
*1*^*st*^ *order Wave load*	*Primary Motion*
*2*^*nd*^ *order wave drift load*	*Superimposed motion*
*Current load*	
*Wind load*	

Another important component of this simulation was the calculation of the drag force for the riser, for which the Morison equation was used. The Morison equation is used to calculate the forces of objects and bodies in harmonic flow, as in Eq (1). *V* is the volume of the body, *A* is the area of the body, D is the diameter of the body, *C*_d_ is the drag coefficient, *C*_a_ is the added mass coefficient, *C*_m_ is the inertial force coefficient, and the *V*_*r*_ is the relative velocity of fluid particles.


$$F=\rho V\dot{u}+\rho C_{a}DA\left(V_{r}\right)+\frac{1}{2}\;\rho C_{d}A(V_{r})\left|V_{r}\right|$$ (1)

## 3. Results and discussion

In this section, the results of global design are presented.

### 3.1. Influence of environmental conditions on bending moment

**Fig 6:**
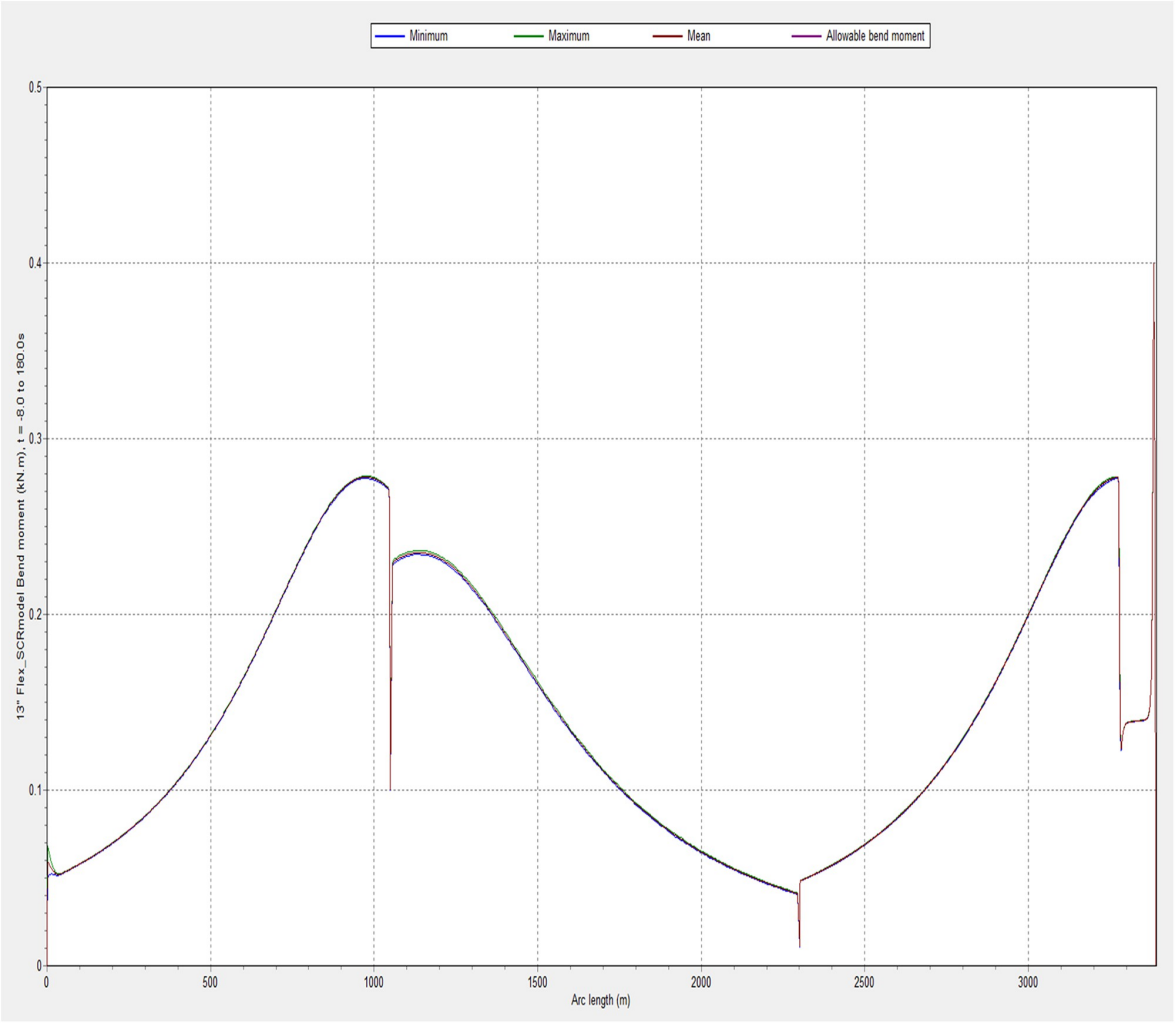
A plot of bending moment for case 1 in OrcaFlex 11.0f.

**Fig 7:**
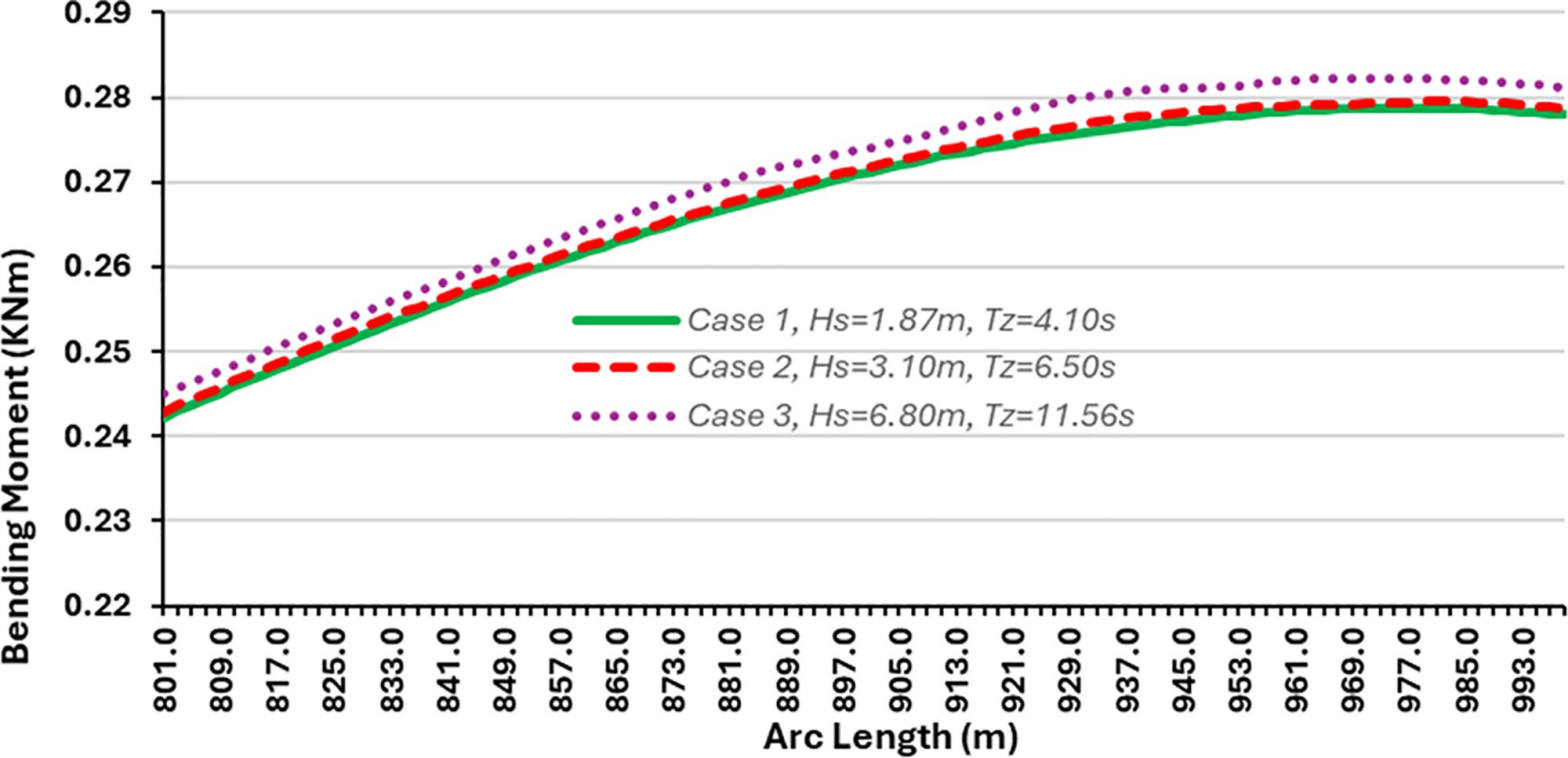
Influence of bending moment for different environmental conditions for arc length 801-993m.

The influence of bending moment was investigated using different environmental conditions. The model was simulated in OrcaFlex using the global design parameters in Section 2. The first simulation was conducted under the least severe conditions. The values selected to configure the ocean environment correspond to the average day-to-day conditions that would be expected for an operational riser. The results demonstrating the effect of the bending moment are presented in Figs 6 and 7. The bending moment in Fig 6 shows sudden changes in the value over small arc length, which shows more like failure or folding, but it is due to the bending stiffness condition in that section of the flexible risers’ arc length. However, further research is required to understand the effect of bending stiffeners on the flexible riser, as it is recommended to have other bending moment plots reflecting the influence of the stiffness. Also, the plotted bending moment in Fig 7 shows value in relation to various environmental conditions, as it shows the significance of the location on for arc length 800-995m, as this section was chosen because it had the maximum bending moment in the result.

As such the values recorded for bending moment, effective tension and shear stress should all lie well within the yield limits of a flexible riser. These types of forces and loads are not anticipated to pose any serious threat to the integrity of the marine riser over a short period. However, these results will be more useful when discussing fatigue and fatigue-induced failures. As the loads seen in this simulation are over a 180-second period, this means that a flexible riser in operation can expect to experience cyclic loading of these magnitudes constantly. Due to the calm sea condition of the ocean environment, the riser displayed very little motion during the 180-second duration of the simulation. This implies that there is minimal bending in that section of the marine riser owing to the tranquil nature of the ocean environment. As illustrated in Fig 7, the greater the wave height, the larger the bending moment.

### 3.2. Influence of environmental conditions on effective tension

**Fig 8:**
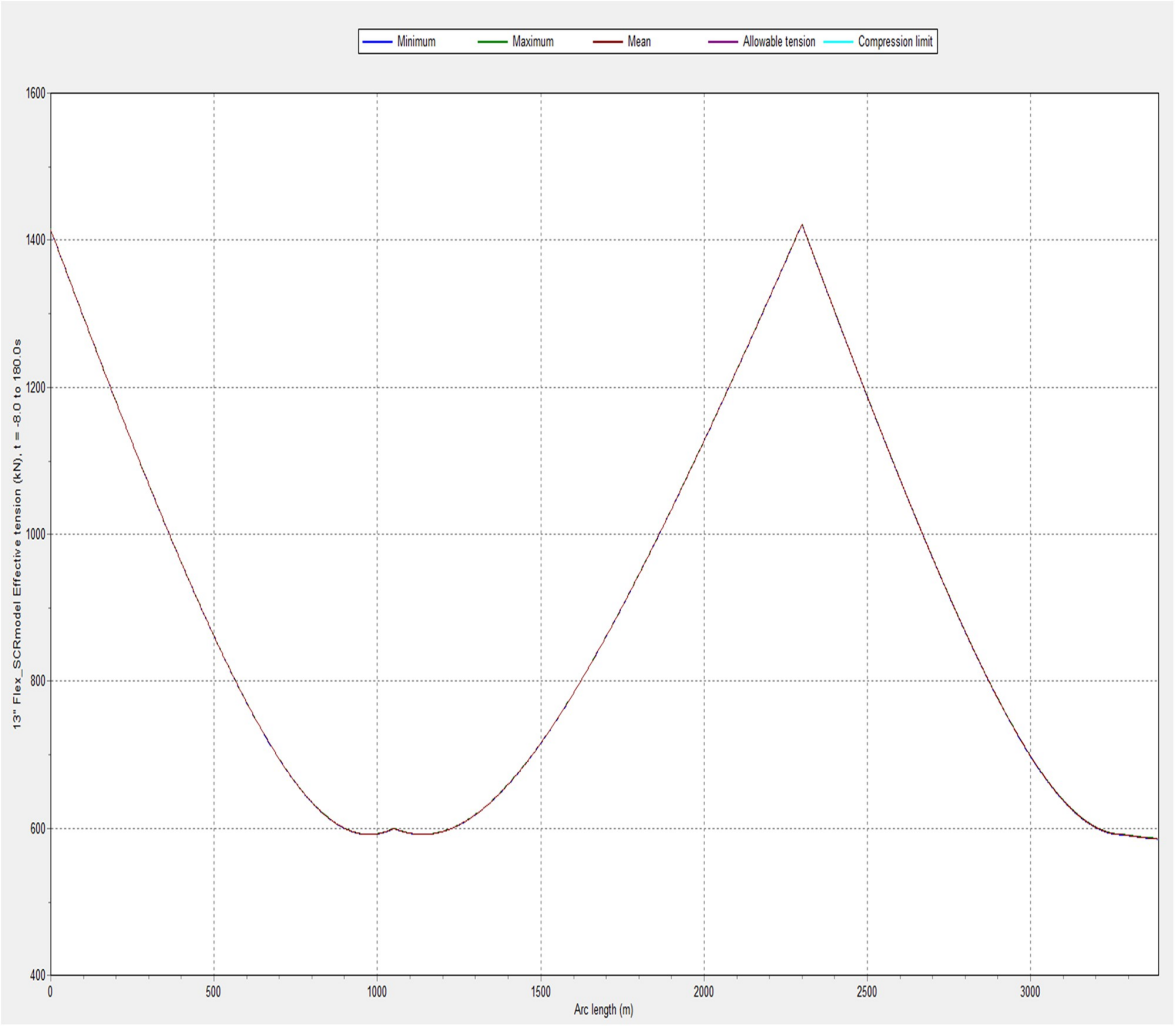
A plot of effective tension for case 1 in OrcaFlex 11.0f.

**Fig 9:**
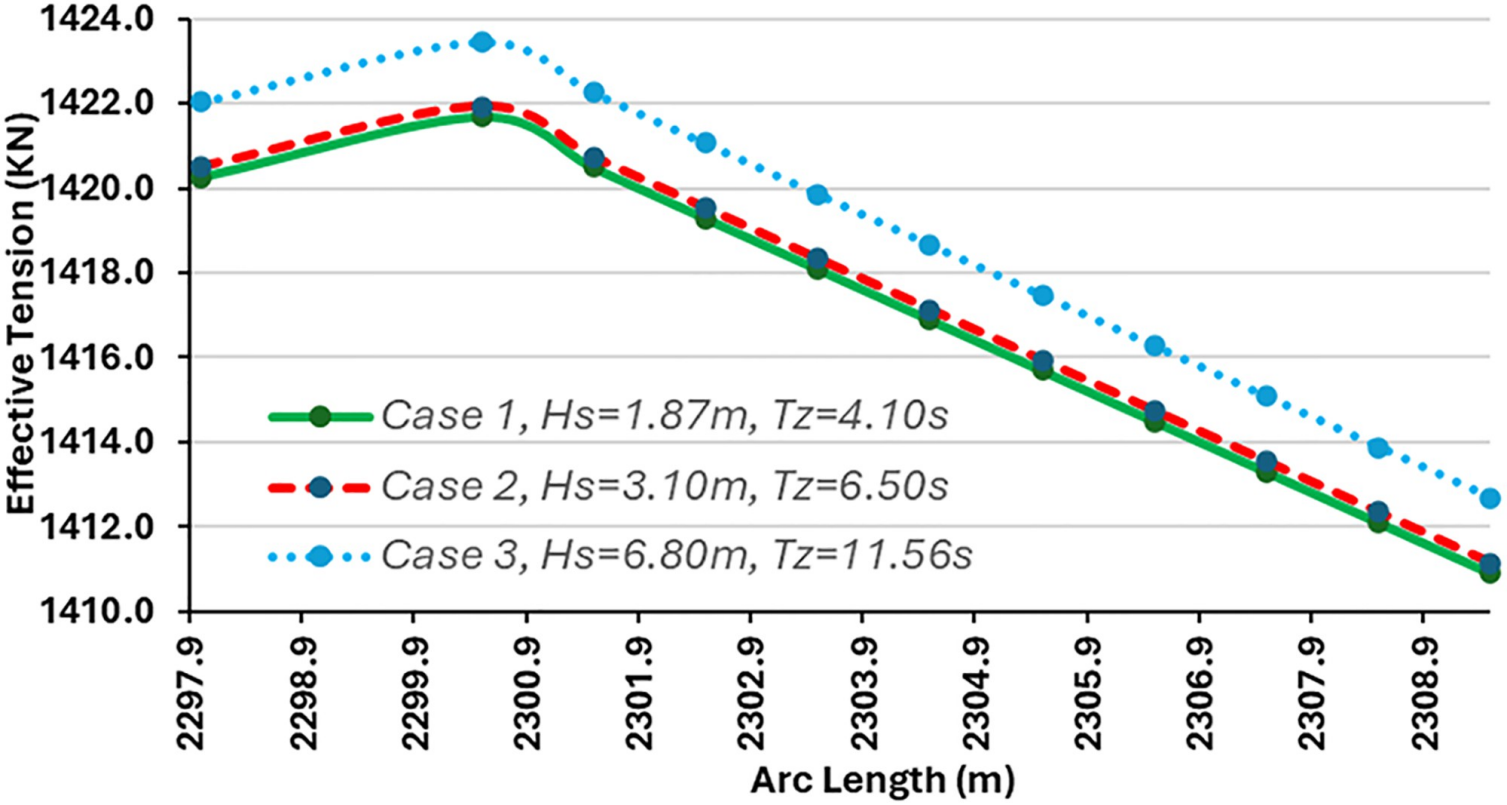
Influence of effective tension on different environmental conditions.

**Fig 10:**
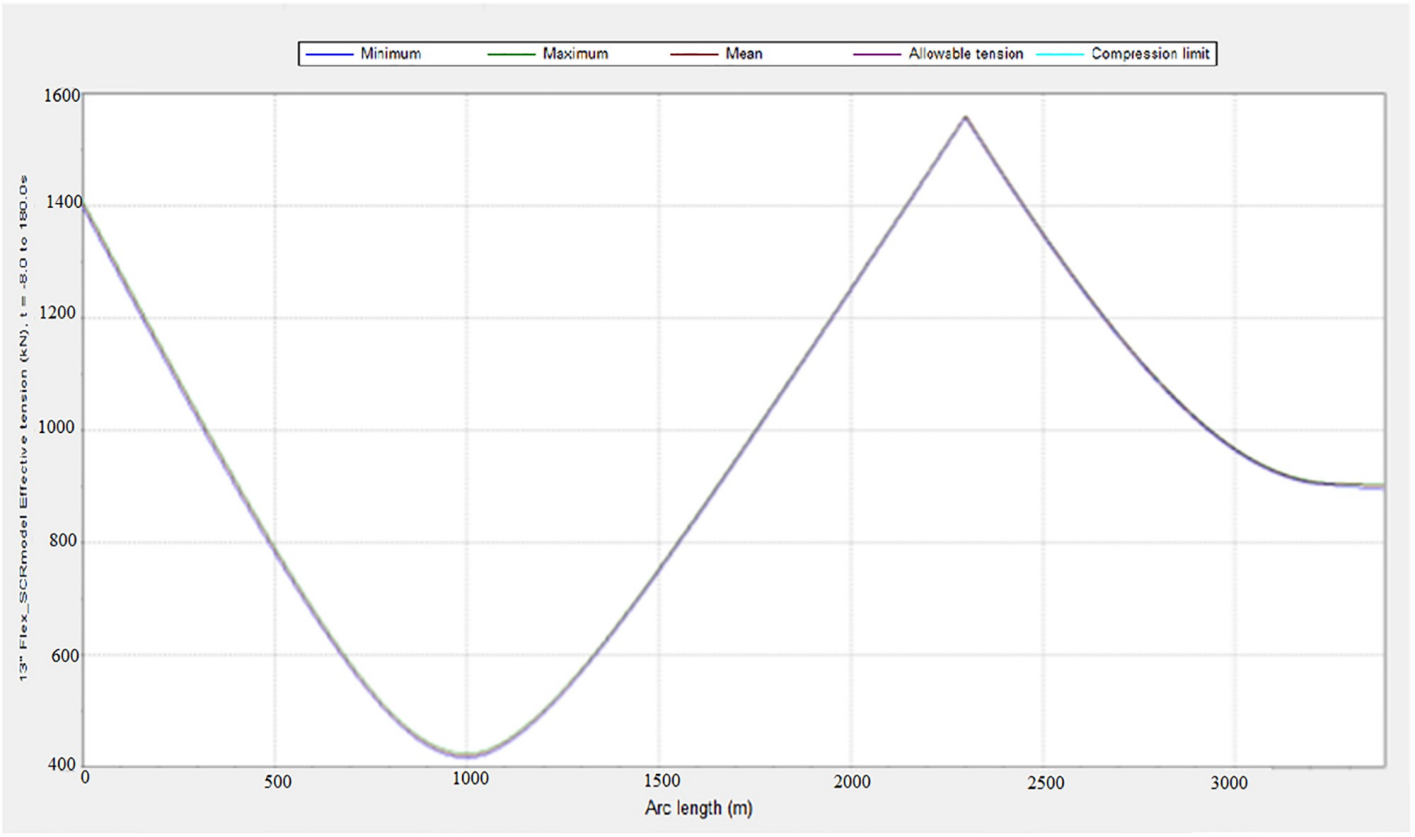
A plot of effective tension for a base case in OrcaFlex 11.0f.

The influence of effective tension was investigated under varying environmental conditions. The model was simulated in OrcaFlex employing the global design parameters in Section 2. It was observed that the variation in the environmental condition also affected the effective tension of the riser system. The configuration of the marine riser included the riser segments and the buoyancy floats. The use of buoyancy floats was found to influence the effective tension. The results depicting the influence of the effective tension are presented in Figs 8–10. As indicated in Figs 9 and 10, the greater the wave height, the larger the effective tension. However, the effective tension at hang-off seems excessive according to the plot in Fig 8, which requires the need to improve the mechanical properties by using bending stiffeners and reducing the length of flexible risers. In addition, the design can be further researched upon by considering the constraints from the limits of the flexible riser used in the study.

### 3.3. Influence of environmental conditions on von Mises stress

**Fig 11:**
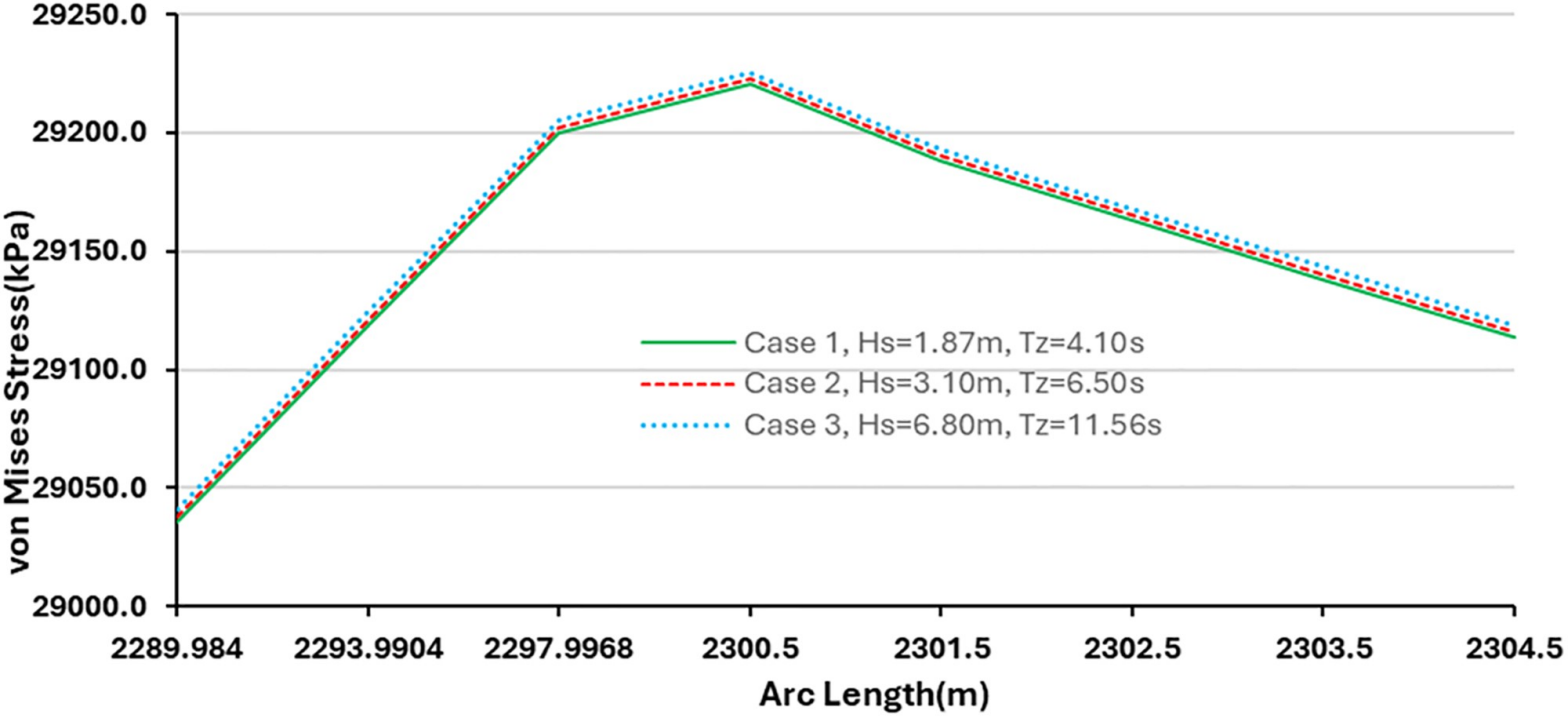
Influence of von Mises stress on different environmental conditions.

The influence of von Mises stress was investigated using different environmental conditions. The model was simulated in OrcaFlex using the global design parameters specified in Section 2. Due to the calm nature of the ocean environment, the riser displayed very little motion during the 180-seconds period of the simulation. The result of the effect of the von Mises stress is given in Fig 11. As indicated in Fig 11, the higher the wave height, the higher the von Mises stress. The exploded view in Fig 11 shows that the stress has an impact on the riser profile, however further investigation is recommended on the finite element modelling of the riser profile. Earlier works on FEM of hybrid composite flexible risers which are available in literature [33, 34, 40], also support that there is the need to further investigate the effect of the layers for the marine risers as well as the orientation.

### 3.4. Result of configuration from riser static analysis

**Fig 12:**
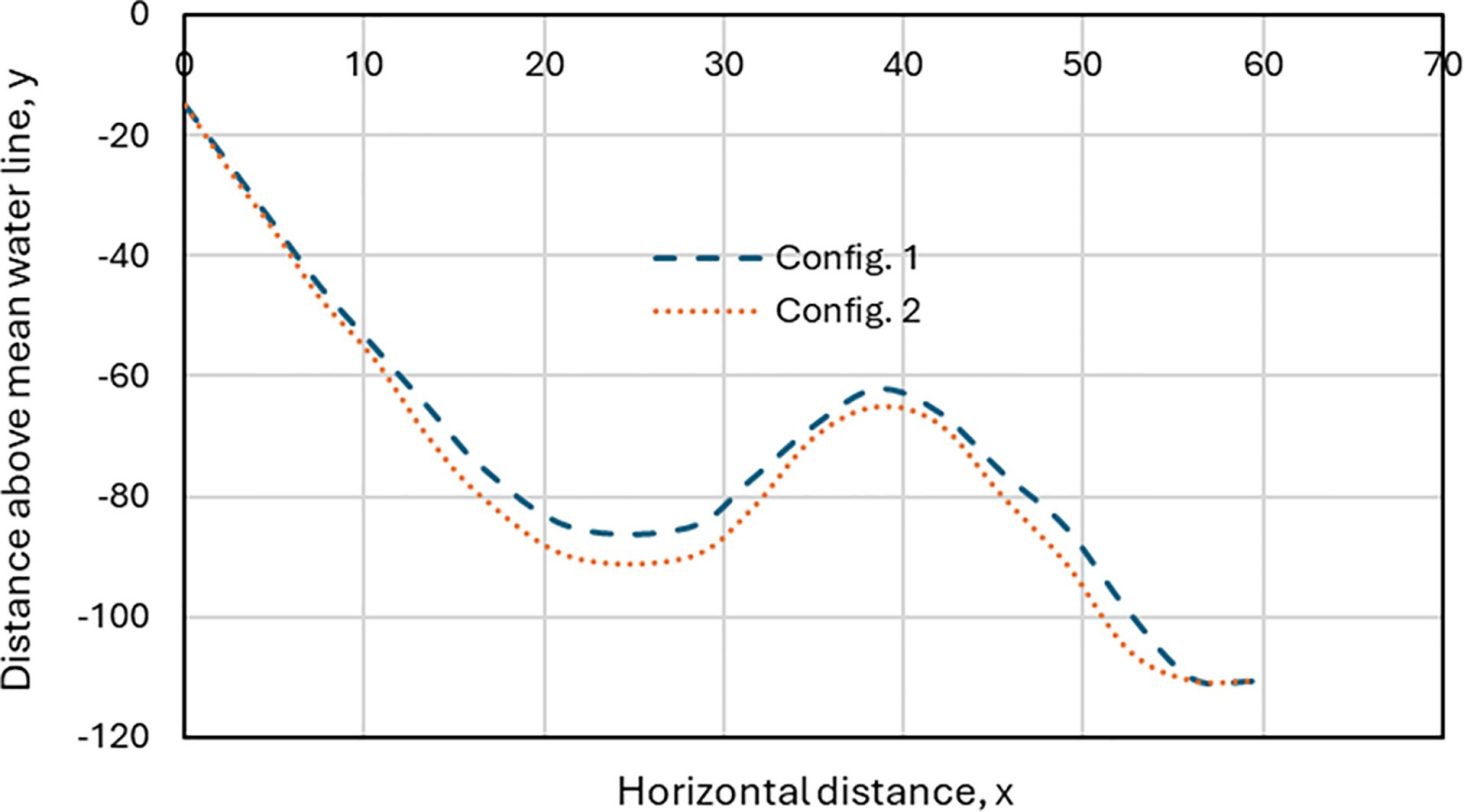
The configuration of the riser from static analysis in OrcaFlex 11.0f.

The influence of the configuration was investigated under static conditions. The static model was used to validate the dynamic analysis model in OrcaFlex software. Two configurations were utilised to understand the behaviour of the flexible riser under waves when attached to an FPSO in ocean conditions. As seen in Fig 12, the behaviour of the riser model depends on the lumped-mass and lumped-stiffness approach of the OrcaFlex line model used. It is also dependent on the buoyancy elements along the arc length of the flexible riser. Various works have presented the mechanics of marine risers for both fixed and floating structures [3–10], which suggest the importance of the static analysis [89–91]. In this study, the curved sections represent maximum and minimum values retrieved from the model, which were used in further dynamic analysis to simulate fully developed motion using the loading history. These findings will lead to a better understanding of the failure modes in marine risers and, as a result, will aid in the prevention of these problems. However, future work is required on the dynamic analysis of this model by coupling other attachments to the system.

## 4. Conclusions

In this study, the design analysis on the armour of a hybrid composite flexible riser under lazy-wave configuration and different environmental conditions was conducted. The objective of this paper is to numerically examine the hybrid flexible risers for usage in offshore applications. The numerical analysis for this model was carried out in OrcaFlex. While this was coupled into the global analysis in ANSYS AQWA R2 2020 with analysis in OrcaFlex 11.0f, this study reported on the aspect conducted on the later tool, as the coupling study is recommended for further work. Another aspect that is recommended for further study include the FEM and optimization models. The reason is that these findings will lead to a better understanding of the failure modes and provide a reference data, for ocean engineering. However, this study provides extensive information on the stresses and forces experienced by various areas of the marine riser. This application is aimed for simulating the dynamic and complex conditions of subsea applications and FPSO attachments.

The following findings are drawn from this study:

1. Different load cases were employed to ascertain the strength behaviour of the hybrid riser. Both the local design and global design were carried out in this paper, and it showed that the design orientation was a factor in the strength behaviour of the riser. The study also showed that the sections with floats had different motion characteristics from the normal bare riser section. This contributes to knowledge, particularly on the mechanics of the hybrid composite flexible risers but more work on the finite element analysis will be required to understand the effect of the layers. Also, future work is required on the dynamic analysis of this model by coupling other attachments to the system.
2. From this study, it was observed that the parameters of the flexible riser are subject to the configuration of the marine riser. Additionally, this application will also assist in simulating the dynamic and complex conditions seen in subsea applications and FPSO attachments. In this study, the results of both the local design and the global design are presented. However, further research is recommended on the global analysis of the hybrid composite flexible risers, underscoring the necessity to consistently reinforce the inner liners by using other composite materials.
3. Furthermore, the motion of the overall system design and implementation is shared by both types of riser structures. Individual sections of both types of risers are constructed, with each riser section typically reaching 10–12 m in length and end-to-end fittings connecting them. The typical ends of this marine risers consist of a metallic flange and a steel nipple, depending on the design. The ends of each segment are bolted together to establish a tight seal between them, which keeps the internal product fluids contained. Hence, further work can be done to investigate the effect of end fittings for this hybrid riser. Also, the mechanical properties (i.e. bending stiffness, axial stiffness, shear strength) of the flexible riser should be utilised to investigate the influence of the bending stiffener.
4. The comparative study based on the parameters considered demonstrated that the influence of static configuration, effective tenson range, stress and bending moment affect the motion performance of the hybrid composite flexible riser along its arc length. However, future study can be conducted on the motion response prediction of the FPSO using machine learning as well as the fatigue performance of the marine riser through optimization techniques. The simulation findings should offer valuable insights into the failure modes and their causes, leading to an enhanced understanding of these modes and, consequently, aiding in the prevention of such issues.
5. The stress results from the hybrid composite flexible riser model provide an understanding of the forces acting on the various segments of the marine riser. However, more comparison is required for structural verification, utilising both the simulation results and other types of analysis. This study offers valuable insights into the failure modes and their causes under static and dynamic systems. Additional studies are recommended on the fatigue investigation of hybrid composite flexible risers and the adaptation of the hybrid composite flexible riser model as a multi-layered marine structure by replacing the metallic alloy liner with a composite liner.

## Supporting information

S1 File (SIM)

S2 File (DOCX)

S3 File (XLSX)

S4 File (ZIP)
