# Supplementary material for: Parametric analysis on the global design of flexible riser under different environmental conditions using OrcaFlex
Source: PLoS One. 2024 Dec 23;19(12):e0310360. doi: 10.1371/journal.pone.0310360 (PMC11666038; doi:10.1371/journal.pone.0310360)
Supplement: S4 File — (ZIP) [file pone.0310360.s004.zip › PONE - Global riser Model-Supplementary files/Permission again for Figure 5 - Ship 6DoF motion.pdf]

Chiemela Victor Amaechi

From: Alberto José Alvarellos González <alberto.alvarellos@udc.es>
Sent: 24 July 2024 08:12
To: Chiemela Victor Amaechi
Subject: Re: Request for permission to reuse and adapt and image
Attachments: vessel_movement.xcf

[This message originated from an External Source]

Hello,

yes I give you permission to use the image.
I'm sending you the original (GIMP format), so you can modify it, would you like to.

Kind regards.

From: Chiemela Victor Amaechi <CAmaechi@globalbanking.ac.uk>
Sent: Tuesday, July 23, 2024 15:54
To: Alberto José Alvarellos González <alberto.alvarellos@udc.es>
Subject: Request for permission to reuse and adapt and image

You don't often get email from camaechi@globalbanking.ac.uk. Learn why this is important

Dear Sir,
I came across one of your publications and would like to reuse and adapt an image. It is one of the images on ship motion for a publication in my research and I will cite the work and also acknowledge your work as the source of the image too. The paper is published as Open Access however, I still need your permission.

The citation for the publication is:
Alvarellos, A.; Figuero, A.; Carro, H.; Costas, R.; Sande, J.; Guerra, A.; Peña, E.; Rabuñal, J. Machine Learning Based Moored Ship Movement Prediction. *J. Mar. Sci. Eng.* **2021**, *9*, 800. https://doi.org/10.3390/jmse9080800

The image is shown here with the following source as "**Figure 2.** Vessels motions along the three axes":

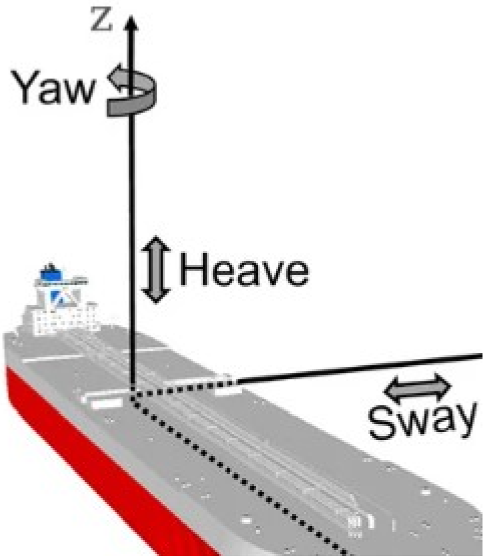

Many thanks,

camaechi@globalbanking.ac.uk

https://globalbanking.ac.uk

GBS
GLOBAL APPLIED KNOWLEDGE

Please consider the environment before printing.

The contents of this email message and any attachments are intended solely for the addressee(s) and may contain confidential and/or privileged information and may be legally protected from disclosure. If you are not the intended recipient of this message or their agent, or if this message has been addressed to you in error, please immediately alert the sender by reply email and then delete this message and any attachments. If you are not the intended recipient, you are hereby notified that any use, dissemination, copying, or storage of this message or its attachments is strictly prohibited.
