# Supplementary material for: Parametric analysis on the global design of flexible riser under different environmental conditions using OrcaFlex
Source: PLoS One. 2024 Dec 23;19(12):e0310360. doi: 10.1371/journal.pone.0310360 (PMC11666038; doi:10.1371/journal.pone.0310360)
Supplement: S4 File — (ZIP) [file pone.0310360.s004.zip › PONE - Global riser Model-Supplementary files/Permission for Figure 5 - Ship 6DoF motion.pdf]

## Chiemela Victor Amaechi

**From:** Andrés Figuero Pérez <andres.figuero@udc.es>
**Sent:** 23 July 2024 21:02
**To:** Chiemela Victor Amaechi
**Subject:** Re: Request for permission to reuse and adapt an image

[This message originated from an External Source]

Dear Chiemala,

I have no problem with you using our image and citing our work.

Good luck in your research.

Kind Regards.

Enviado desde Outlook para Android

---

**From:** Chiemela Victor Amaechi <CAmaechi@globalbanking.ac.uk>
**Sent:** Tuesday, July 23, 2024 3:53:43 PM
**To:** Andrés Figuero Pérez <andres.figuero@udc.es>
**Subject:** Request for permission to reuse and adapt an image

No suele recibir correos electrónicos de camaechi@globalbanking.ac.uk. Por qué esto es importante

Dear Sir,
I came across one of your publications and would like to reuse and adapt an image. It is one of the images on ship motion for a publication in my research and I will cite the work and also acknowledge your work as the source of the image too. The paper is published as Open Access however, I still need your permission.

The citation for the publication is:
Alvarellos, A.; Figuero, A.; Carro, H.; Costas, R.; Sande, J.; Guerra, A.; Peña, E.; Rabuñal, J. Machine Learning Based Moored Ship Movement Prediction. *J. Mar. Sci. Eng.* **2021**, *9*, 800. https://doi.org/10.3390/jmse9080800

The image is shown here with the following source as "**Figure 2.** Vessels motions along the three axes":

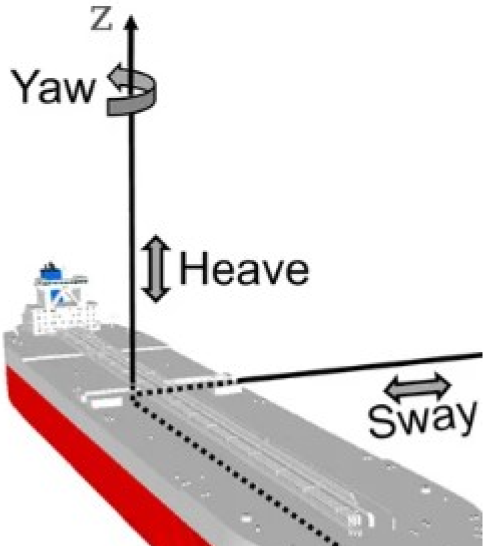

Lecturer in Construction Management | Cohort Lead Oct 22
GBS Manchester Campus | Bath Spa University partnership

camaechi@globalbanking.ac.uk

https://globalbanking.ac.uk

GBS
GLOBAL APPLIED KNOWLEDGE

**Please consider the environment before printing.**

The contents of this email message and any attachments are intended solely for the addressee(s) and may contain confidential and/or privileged information and may be legally protected from disclosure. If you are not the intended recipient of this message or their agent, or if this message has been addressed to you in error, please immediately alert the sender by reply email and then delete this message and any attachments. If you are not the intended recipient, you are hereby notified that any use, dissemination, copying, or storage of this message or its attachments is strictly prohibited.
