# Supplementary material for: Parametric analysis on the global design of flexible riser under different environmental conditions using OrcaFlex
Source: PLoS One. 2024 Dec 23;19(12):e0310360. doi: 10.1371/journal.pone.0310360 (PMC11666038; doi:10.1371/journal.pone.0310360)
Supplement: S4 File — (ZIP) [file pone.0310360.s004.zip › PONE - Global riser Model-Supplementary files/PLoS ONE - Method description.docx]

**Supplementary File - The Method used in the study:**

Firstly, we identified the title for the paper, then carried out a brief literature review. It was then followed with fundamental understanding of the hybrid riser system under consideration.
Then we started to model the marine riser in Orcaflex using a configuration that will suit the design, the water depth and our solution.

Bearing in mind that the problem involves modelling a hybrid riser structure that can withstand the wave forces and other loadings in a deepwater condition. The research involved the use of ANSYS AQWA as well, but this aspect presented mainly the works in Orcaflex, as these results are based on the parameters investigated.

To understand the configuration, we started with a typical geometry model for the work and considered some theoretical understanding (see Figure 1). The analysis of the results were also another aspect of the research that involved some industry understanding. While we used an FPSO to conduct the model, the findings did reflect the challenges found in the field. However, the researchers did not have the resources of conducting sea trials on the research.


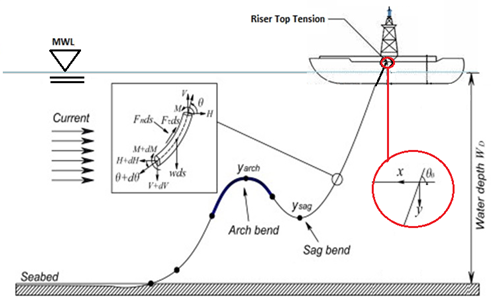


**Figure 1**. Typical marine riser configuration showing forces for a differential element

Supplementary files added include:

Permissions received for Figure 5 in the article, the original file for the vessel motion, the original Orcaflex file used, the method description file, and the result spreadsheet file.
