# Supplementary figures and images for: Parametric analysis on the global design of flexible riser under different environmental conditions using OrcaFlex
Source: PLoS One. 2024 Dec 23;19(12):e0310360. doi: 10.1371/journal.pone.0310360 (PMC11666038; doi:10.1371/journal.pone.0310360)

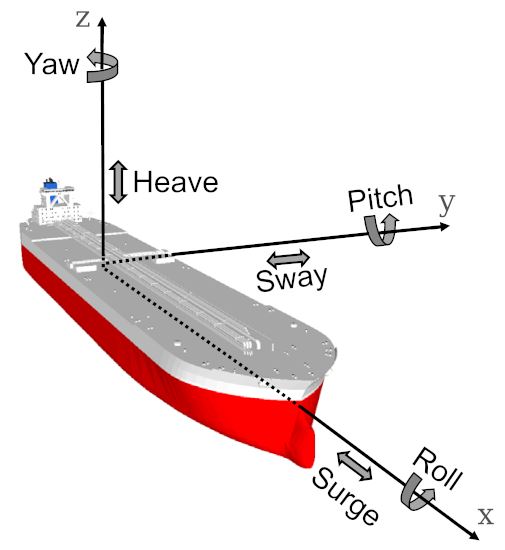

Supplement: S4 File — (ZIP) [file pone.0310360.s004.zip › PONE - Global riser Model-Supplementary files/Ship Motion - Figure 5 -Alvarellos et al. 2021.JPG]
